# The effect of juvenile systemic lupus erythematosus serum activated macrophages on a lupus nephritis model

**DOI:** 10.1186/1546-0096-12-S1-P113

**Published:** 2014-09-17

**Authors:** April S Vernon, Angela Midgley, Michael Beresford

**Affiliations:** 1 Child Health, Translational Medicine , University of Liverpool, UK

## Introduction

Podocytes are kidney cells residing in the Bowman’s capsule wrapping around the capillaries of the glomerulus; they form a seal, which contributes greatly to the glomerular filtration barrier (GFB). Their function is highly linked to their morphology, alterations result in loss of integrity of the podocyte seal which can allow the diffusion of proteins from the circulation resulting in proteinuria. A human podocyte cell-line with human-monocyte derived macrophages can be used to create an in vitro model of lupus nephritis (LN). Using this model, it is known that the chemokine monocyte chemoattractant protein 1 (MCP-1) may be released, which has also been identified as a biomarker of the active disease. MCP-1 causes recruitment of pro-inflammatory cells to the kidney. Incubation of this model with JSLE serum may mediate an inflammatory response representing a biologically relevant environment and subsequently provide a clearer understanding into the development of LN.

## Objectives

To determine the effect of JSLE serum on the regulation of MCP-1 produced in a LN in *vitro* model.

## Methods

Human monocytes were differentiated into macrophages using media containing monocyte colony stimulating factor. The macrophages were then either unstimulated (inactive cells) or incubated with IFN-γ (1ng/ml) or 5% JSLE serum. After 48 hours the supernatant from the macrophages were removed and added to mature podocytes. MCP-1 concentration (mean±SEM) was measured using ELISA. Images were taken using a light microscope to allow any morphological changes in the podocytes to be observed.

## Results

Previously we have shown IFNγ activated macrophages to produce MCP-1 which in turn increases the concentration of MCP-1 produced by podocytes, while unstimulated macrophages show no difference. Importantly when JSLE serum (n=4) is added to unstimulated macrophages, it activates them in a similar manner to IFNγ alone. Incubated with JSLE serum elicited a significant increase in MCP-1 concentration produced by the macrophages (1357±84ng/ml) compared with unstimulated (252±162ng/ml; p=0.0001). There was no significant difference in MCP-1 concentration compared to IFNγ activated macrophages (1396±349ng/ml; p=0.477). When JSLE serum stimulated macrophages are co-cultured with podocytes, MCP-1 concentration is again found to be significantly higher than unstimulated macrophages (2567±87ng/ml; p=0.0002) with no significant difference with IFNγ activated macrophages (3030±289ng/ml; p=0.099). Podocytes incubated with JSLE serum activated macrophages also result in dramatic changes to the podocyte morphology which was again similar to that seen with IFNγ activated macrophages.

## Conclusion

JSLE serum stimulated the macrophages in an analogous way to IFNγ, resulting in increased MCP-1 production by both the macrophages and podocytes, suggesting that macrophage activation by IFNγ is similar to that observed in LN. However, the concentration of MCP-1 does not exceed that which is produced by the co-culturing of podocytes with IFNγ activated macrophages. Therefore serum factors directly acting on the podocytes need to be identified. The importance of IFN’s in JSLE pathogenesis is widely accepted however the role of the IFN subtypes in the development of the disease is unclear. This study provides evidence to suggest that factors in JSLE serum such as IFNγ may play a prominent role in the activity and progression of the disease particularly when there is kidney involvement.

## Disclosure of interest

None declared.

